# Emerging Hepatotropic Viruses in Cats: A Brief Review

**DOI:** 10.3390/v13061162

**Published:** 2021-06-17

**Authors:** Paolo Capozza, Nicola Decaro, Farzad Beikpour, Canio Buonavoglia, Vito Martella

**Affiliations:** Department of Veterinary Medicine, University of Bari Aldo Moro, 70010 Valenzano, BA, Italy; paolo.capozza@uniba.it (P.C.); nicola.decaro@uniba.it (N.D.); farzadbeikpour@yahoo.it (F.B.); canio.buonavoglia@uniba.it (C.B.)

**Keywords:** cat, hepatotropic viruses, hepadnaviruses, hepeviruses

## Abstract

The possible role of viruses in feline liver disease has long remained neglected. However, in 2018, an analogue of human hepatitis B virus was identified in cats. Moreover, antibodies for human hepatitis E have been detected consistently at various prevalence rates in cats. Although the correlation between these viruses and the liver injury in cats must be clarified, hepatotropic viruses might represent an increasing risk for feline and public health.

## 1. Introduction

The liver plays a central homeostatic role within the body, being involved in several metabolic pathways. In addition to metabolic functions, the liver is the site of synthesis of albumin and the majority of clotting factors. The liver functional position, between the gastrointestinal tract and the systemic circulation, is accounted for by its key role in digestion, detoxification, and immune surveillance. All of the features of liver function have an important impact on the clinical presentation of animals with liver disease [1]. The early clinical signs typically observed in liver disease include intermittent fever, anorexia, polyuria/polydipsia, vomiting, diarrhea, lethargy, weight loss, and abdominal pain, all of which are nonspecific signs. More specific clinical signs, such as jaundice or ascites, tend to occur later in the disease process [1,2]. 

In general, the liver enzymes, as alanine aminotransferase (ALT), aspartate aminotransferase (AST) alkaline phosphatase (ALP) and gamma-glutamyl transferase (GGT), are sensitive indicators of liver disease or injury but are not specific and do not offer any precise indication of liver functionality [1,3]. In cats, even a small increase in ALT is considered an indicator of liver injury, due to the limited serum half-life of ALT, and an increase in AST could imply significant liver injury, due to its mitochondrial localization [3].

Along with hepatotropic viruses primarily associated with hepatitis (hepatitis A to E) and regarded as the major agents of hepatitis, in humans a number of other viruses can replicate in the hepatic tissues and cause hepatitis secondarily after systemic infection, including herpesviruses (cytomegalovirus, Epstein-Barr virus, human herpesvirus 6), parvovirus B19 and picornaviruses [4]. Likewise, in cats several infectious agents may affect the liver either primarily or in course of systemic infection. Although infections by common bacteria are probably the most common cause of infectious hepatitis in cats, other potential microorganisms, including mycobacteria, viruses, fungi, protozoa, parasites, and rickettsiae can cause liver injury [2]. 

Viruses associated with liver impairment include feline leukemia virus (FeLV), feline infectious peritonitis virus (FIPV) and hypervirulent feline calicivirus strains associated with virulent systemic disease (FCV-VSD) [2,5]. FeLV, a retrovirus of the genus *Gammaretrovirus*, replicates in several feline tissues, with the clinical illness being generally related to the involvement of hematopoietic and immune systems. FeLV has been also associated with icterus and various inflammatory and degenerative liver diseases including focal liver necrosis [6,7,8,9]. FIPV is a hypervirulent feline coronavirus that results from genomic RNA mutations triggering the ability to enter and replicate in macrophages [10,11]. An immune mediated vasculitis occurs if the virus is not eliminated. Affected cats developed signs related to the target organ lesions (kidney, liver, central nervous system, intestine) or due to fluid redistribution. Abnormal liver enzymes can occur because of hepatitis, hepatic lipidosis, or prehepatic sequalae of vasculitis, erythrocyte destruction, and hypoxia. Hyperbilirubinemia is common and usually secondary to vasculitis in the liver [12]. Histopathology is required for definitive diagnosis but it can be supported by history, physical examination, and laboratory findings [12]. 

FCV-VSD, which includes highly virulent variants of FCV, can have mortality rates of up to 67% in adult cats [5,13]. Primarily identified in shelter or cattery populations, these FCV variants cause profound fever, anorexia, marked subcutaneous edema (limbs and face especially), jaundice, alopecia, crusting and ulceration of the nose, lips, ears and feet [13,14,15]. Adult cats are most severely affected. Individual hepatocyte necrosis ranging to centrilobular, or more extensive necrosis is associated with neutrophilic inflammatory foci and intrasinusoidal fibrin deposits [16]. 

Recently, the presence of hepatotropic viruses, belonging to the viral families Hepadnaviridae and Hepeviridae has been reported in cats either directly or indirectly. This has raised interests in human and veterinary research since hepadnaviruses and hepeviruseses are well established human pathogens. The aim of this review is to provide a general overview of emerging hepatotropic viruses in cats, in order to draw the attention of veterinary clinicians, microbiologists, and public health officials to these viruses that could be relevant for feline medicine and public health.

## 2. Domestic Cat Hepadnavirus (DCH)

Domestic cat hepadnavirus (DCH) (genus *Orthohepadnavirus*, family Hepadnaviridae), recently identified in cats, is a small (42–50 nm in diameter), partially double-stranded, circular, DNA virus, belonging to the same family as human hepatitis B virus (HBV), a major human pathogen, which leads to chronic infections with increased risk of liver disease, including cirrhosis and hepatocellular carcinoma (HCC) [17,18,19]. DCH has a genome of approximately 3.2 kb of DNA, which similar to other hepadnaviruses, has four overlapping open reading frames (ORFs) encoding for the polymerase (L), surface (S), core (C) and X proteins. Genome sequences derived from DCH-positive domestic cats in Australia [18], Italy [20], and Malaysia [21], show 97.0–98.3% nucleotide similarity to each other. Recently a putative DCH recombinant strain was found in Thailand, suggesting a possible role of recombination in DCH evolution [22].

DCH was first identified in 2018, in Australia, from a transcriptomic study that aimed to analyze alterations in gene expression in a seven-year-old male neutered domestic shorthair cat, presenting with vomiting and weight loss, and diagnosed with multicentric large B cell lymphoma and concomitant infection with feline immunodeficiency virus (FIV) [18]. 

Subsequent screenings with molecular assays (degenerate PCR and real time PCR), carried out by independent research groups around the world, have revealed positivity rates of 6.5% (six in 54) in Australia [18], 10.8% (42 in 390) in Italy [20], 12.4% (26 in 209) in Thailand [22], and 12.3% (31 in 253) in Malaya [21] in whole blood and serum samples. Moreover, DCH DNA has been detected in liver tissue [21,23], with a higher positivity rate (14%; 13 in 84) than in blood/serum [21]. The formation and maturation of DCH particles in the nucleus and/or cytoplasm of infected hepatocytes suggests that hepatocytes are the initial localization of the virus as described in HBV infection [22]. 

DCH has been associated with feline retroviruses [18,20,21,22], since DCH prevalence is higher in FIV co-infected cats [21]. Since feline retroviruses cause impairment of the immune system [7,8], the detection of DCH in cats with retroviral infection could be related to the immunocompromised status, as observed for HBV [24,25]. The transmission of HBV in humans occurs through blood and other body fluids, including serum, saliva, and semen, and transmission can also occur during sexual contacts and by maternal/fetal route [17,26]. Transmission of FIV/FeLV in cats occurs with similar modalities, as the virus is present in blood and body fluids [7,8,9]. Since DCH can be detected in serum and whole blood samples [18,20,21,22], similar modalities of transmission might also be hypothesized for DCH transmission [20,26]. However, in a longitudinal observational study in two cats naturally co-infected with DCH and feline retroviruses, oral, conjunctival, preputial, and rectal swabs repeatedly tested negative to DCH [26]. Other risk factors for DCH infection are still unclear. Cats older than two years have an approximately two-fold higher risk of DCH infection than younger cats [21]. In contrast, breed and gender are not risk factors for DCH infection, although male cats displayed higher prevalence rates than females [20,21]. Further investigations on the possible association of DCH with others risk factors is needed to elucidate the epidemiology of this virus. 

A correlation has been observed between DCH infection and suspected clinical signs of liver injury [20,26]. Cats with DCH vireamia are likely to have elevated ALT values [20,21]. Cats with hematochemical profile suggestive of liver injury have approximately 3-fold higher odds to be DCH positive than cats with haematochemical parameters within physiological ranges [21]. This finding indirectly supported the possible role of DCH in the development of feline liver disease, similar to HBV infection in humans [17]. Chronic HBV infection can trigger immune-mediated chronic hepatitis that is characterized by necrosis, regeneration, and fibrosis, progressing to cirrhosis and HCC [27]. The pathogenesis of HBV-associated hepatopathies and the interaction between HBV and the diverse risk factors has not been completely understood [17]. Chronic hepatitis and HCC may also occur in domestic cats, and are usually considered idiopathic in this species [28]. In an international, multicenter investigation carried out in collaboration with feline medicine specialists from USA, UK, Australia, and New Zealand, the possible role of DCH in chronic hepatitis and HCC has been investigated. The histopathologic features of inflammation and neoplasia, as well as the viral distribution on in situ hybridization (ISH), were strikingly similar to those seen in HBV-associated disease [23]. Several microscopic features that are consistent with, but not pathognomonic for, human HBV-associated hepatitis, including “piecemeal” necrosis, apoptotic bodies, and sinusoidal inflammation [29], have been identified in cases of DCH-associated chronic hepatitis but not in DCH-negative hepatitis. Similarly, DCH-associated HCC shares features with hepatopathies caused by HBV and/or woodchuck hepatitis virus (an orthohepadnavirus of *Marmota monax*), including regions of vacuolar change and individual hepatocellular necrosis. In HCC cases, hepatocyte proliferation in DCH-positive liver areas was greater than in DCH-negative areas. This finding is also described for HBV, since hepatocyte proliferation driven by the host immune response contributes to hepatocyte transformation in HBV-associated HCC [30]. Accordingly, HBV-associated HCC usually arises from a background of chronic inflammation and cirrhosis [31]. 

Chronic hepatitis has been reported to occur with a frequency of 2.4% of all feline liver biopsies [28]. Primary hepatic neoplasm is estimated to represent 1–2.9% of all cancers [32]. Biliary carcinomas and HCC have been reported with a frequency of 17% and 27% of all feline liver malignancies [28]. In a multicentric study, HCC was the most common primary epithelial tumor, with a frequency of 54%, whereas DCH viremia has been detected in over 10% of the general cat population [23]. 

Evidence for hepadnavirus replication in cells other than hepatocytes comes from studies carried out in ducks infected with duck hepatitis B virus (DHBV) and woodchucks chronically infected with orthohepadnaviruses [17]. 

Although in patients with HBV chronic hepatitis, HBV has been identified in various cell types [17,33,34,35], HBV replication seems restricted to the liver tissues. Viral entry in the hepatocellular cells is mediated by the NTCP receptor [36,37,38].

In infected cats, DCH has been identified in the liver and in various extra-hepatic tissues and cells [22]. DCH infection has been revealed in cats with glomerulonephritis in the renal and vascular endothelial cells, and the authors speculated on the possible association between DCH and glomerulo-nephropathy, assuming that kidney tissues are permissive for DHC replication, as hypothesized in HBV infection [22]. Moreover, DCH antigens have been identified in neuron and neuroglia cells, suggesting the ability of DCH to cross the blood brain barrier and replicate in the nervous tissues [22]. Overall, the active replication of DCH in extrahepatic tissues remains to be demonstrated. 

Prospective studies are necessary to establish the geographical distribution and evolution of this HBV-like virus in cats, as well as its pathobiology and possible involvement in the progression of liver disease from inflammation to neoplasm.

## 3. Hepatis E Virus (HEV)

Hepatis E virus (HEV) (Hepeviridae family) one of the five majors human hepatotropic viruses [39], is a small (27-34 nm), non-enveloped virus with an icosahedral capsid. The HEV genome is a single-stranded, positive-sense, polyadenylated RNA molecule of approximately 7.2 kb in length, excluding the poly (A) tail [40,41]. The genome contains three open reading frames (ORFs), a 5’-methylguanine cap and 3’ poly (A) stretch, as well as two 5’ (27 to 35 nucleotides) and 3’ (67 nucleotides) untranslated regions (UTRs) [40,41,42,43]. Hepeviruses are classified into two genera, namely *Piscihepevirus* and *Orthohepevirus*. The latter includes the species *Orthohepevirus A, B, C, and D*. The species *Orthohepevirus A* (HEV-A) comprises eight genotypes that infect human (Gt 1, 2, 3, 4, and 7) and animals (Gt 3, 4, 5, 6, 7, 8). *Orthohepevirus B* consists of avian viruses and is divided into four proposed subtypes (I–IV). *Orthohepevirus C* (HEV-C) includes two genotypes mainly detected in rats (HEV-C1) and carnivores (HEV-C2) [41]. Although the zoonotic potential of HEV-C is still under debate, clinical cases of persistent hepatitis in a liver transplant patient in Hong Kong and of severe acute hepatitis in an immunocompetent patient in Canada have been related to infection with HEV-C of rodents [44,45]. Furthermore, an additional seven cases of human infection with rodent-derived HEV-C have been reported in Hong Kong [44]. *Orthohepevirus D* strains have been detected in different bat species [46,47].

Humans are the natural host of Gts 1 and 2, whilst the other Gts are zoonotic. The clinical forms range from asymptomatic infections to mild-to-moderate liver dysfunction and to fulminant hepatitis [48,49,50]. Persistent hepatitis E can develop in immunocompromised individuals, which can progress to liver cirrhosis if left untreated [51,52]. The HEV-A transmission pathways include the fecal-oral route with zoonotic foodborne origin, person-to-person, blood transfusion, solid organ transplantation, nosocomial, vertical, and direct zoonotic transmission [53]. Currently, pigs, wild boars, and deer are recognized as the main HEV-A reservoirs for human infection [54,55,56], and the consumption of raw or undercooked pork meat and liver has been recognized as the common source of infection in developed countries [57,58]. 

Serological and molecular screening have documented HEV infection in several other animal species providing new insights into the epidemiology of hepatitis E [55]. Anti-HEV-A antibodies have been detected in several animal species other than swine, such as rodents, cows, sheep, goats and dogs and cats [59,60,61,62]. Although anti-HEV-A globulins and HEV-A RNA have been detected in wild and domestic carnivores [61,62,63,64], it is unclear if dogs and cats may develop clinical illness after infection with HEV-A or transmit the virus to the human host [61,62,63,64,65,66,67,68,69,70]. The large differences in the diagnostic methods used for the identification of HEV-A infection and the size and planification of the surveillance studies in the various animal hosts make difficult to compare the data. Indeed, in several studies, only HEV-A-specific antibodies were detected, whilst virus RNA was not identified [61,62,63,64,67,68,70,71,72]. Moreover, eventual cross-antigenic reactivity between HEV-A and non-HEV-A viruses in the serological investigations were not ruled out.

Cats were first suggested to be a potential source of HEV infection for humans in 2003, in Japan, following a sporadic acute case of hepatitis E in a 47-year-old man whose pet cat tested positive serologically to HEV [71]. A serosurvey performed in cats from five major metropolitan areas of China during 2012–2013 reported an overall HEV seroprevalence of 6.28% (12 in 191) [63]. By molecular and serological surveillance conducted over a five-year period (from 2000 to 2004) in cats and dogs in Japan, a seroprevalence for HEV-A of 1.9% was observed in cats [62]. In contrast, in another Japanese study, the seroprevalence of HEV-A in cats was as high as 33% [61]. 

Serological studies in cats in Europe have revealed prevalence rates of 3.1% (10 in 324) in Italy [72], 11.0% (six in 54) in Spain [68], 14.9% (seven in 47) in The Netherlands [70] and 32.3% (21 in 65) in Germany [64]. These findings indicate that HEV-like viruses circulate in the feline population worldwide. However, since only antibodies have been detected so far, the exact nature of these serological positivities remains to be investigated. Moreover, it remains to be clarified if HEV-like viruses are commonly harbored in cats or they are incidentally transmitted from other mammals acting as HEV reservoirs for cats.

Currently, there is no evidence for acute and chronic liver injury in cats being correlated to the presence of HEV antibodies or HEV RNA. Risk factors for HEV infection in cats could be represented by feline lifestyle and dietary habit conditions. A much higher positive rate has been reported in pet cats (17.6%, six in 34) consuming food waste than in cats fed on commercial food (3.8%, six in 157) [63]. A possible source of infection for domestic and wild carnivores could be represented by the ingestion of animals recognized as HEV reservoirs, such as rats, although further investigation is needed to confirm this hypothesis. 

Evidence for the transmission of Gt3 and 4 by direct contact of humans with animals has been repeatedly described, although in most cases the clinical consequences are not clear. Several studies have shown that persons with occupational contact to domestic pigs such as slaughterers, pig farmers or veterinarians exhibit significantly higher anti-HEV antibody prevalence than the control population [73,74,75,76]. However, whether pet veterinarians and pet owners are at higher risk of HEV exposure has not been determined [69,76,77]. Moreover, the discovery of the zoonotic potential of HEV-C [44,45] increases the interest for HEV-like viruses, making it necessary to better understand the ecology of these zoonotic viruses. Likewise, further studies are needed to assess the risks to public health presented by human contact with domestic cats.

## 4. Conclusions

In cats, several infectious agents including viruses can affect the liver primarily or secondarily during the course of systemic infection. In addition, novel hepatotropic viruses, i.e., DCH and HEV, related or similar to viruses causing liver injury in the human host, have been detected or suspected. Considering the popularity of cats as companion animals, the potential impact of DCH and HEV on feline and public health could pose challenges for the global veterinary community. For instance, the possibility of long-term (chronic) DCH infections in cats may have implications for transfusion medicine, requiring an update of the diagnostic algorithms used to ensure the safety of blood donors.

## Data Availability

Not applicable.

## References

[1] O’Neill E., David B. (2020). Approach to the Patient with Liver Disease. Clinical Small Animal Internal Medicine.

[2] Kearns S. (2009). Infectious hepatopathies in dogs and cats. Top. Companion Anim. Med..

[3] Scott S.L.S.M.A. (2008). Enzymes. Fundamentals of Veterinary Clinical Pathology.

[4] Margolis H.S., Alter M.J., Hadler S.C., Evans A.S., Kaslow R.A. (1997). Viral Hepatitis. Viral Infections of Humans.

[5] Gaskell R.M., Susan D., Alan R., Greene C.E. (2012). Feline Respiratory Disease. Infectious Diseases of the Dog and Cat.

[6] Reinacher M., Theilen G. (1987). Frequency and significance of feline leukemia virus infection in necropsied cats. Am. J. Vet. Res..

[7] Hartmann K. (2012). Clinical aspects of feline retroviruses: A review. Viruses.

[8] Hartmann K. (2011). Clinical aspects of feline immunodeficiency and feline leukemia virus infection. Vet. Immunol. Immunopathol..

[9] Hartmann K., Greene C.E. (2012). Feline Leuckemia Virus Infection. Infection Diseases of Dog and Cat.

[10] Decaro N., Lorusso A. (2020). Novel human coronavirus (SARS-CoV-2): A lesson from animal coronaviruses. Vet. Microbiol..

[11] Decaro N., Mari V., Lanave G., Lorusso E., Lucente M.S., Desario C., Colaianni M.L., Elia G., Ferringo F., Alfano F. (2021). Mutation analysis of the spike protein in Italian feline infectious peritonitis virus and feline enteric coronavirus sequences. Res. Vet. Sci..

[12] Addie D.D., Greene C.E. (2012). Feline Coronavirus Infections. Infectious Diseases of the Dog and Cat.

[13] Radford A.D., Addie D., Belak S., Boucraut-Baralon C., Egberink H., Frymus T., Gruffydd-Jones T., Hartmann K., Hosie M.J., Lloret A. (2009). Feline calicivirus infection. ABCD guidelines on prevention and management. J. Feline Med. Surg..

[14] Radford A.D., Coyne K.P., Dawson S., Porter C.J., Gaskell R.M. (2007). Feline calicivirus. Vet. Res..

[15] Caringella F., Elia G., Decaro N., Martella V., Lanave G., Varello K., Catella C., Diakoudi G., Carelli G., Colaianni M.L. (2019). Feline calicivirus infection in cats with virulent systemic disease, Italy. Res. Vet. Sci..

[16] Pesavento P.A., MacLachlan N.J., Dillard-Telm L., Grant C.K., Hurley K.F. (2004). Pathologic, immunohistochemical, and electron microscopic findings in naturally occurring virulent systemic feline calicivirus infection in cats. Vet. Pathol..

[17] Seeger C., Zoulim F., Mason W.S., Knipe D.M. (2013). Hepadnaviruses. Fields Virology.

[18] Aghazadeh M., Shi M., Barrs V.R., McLuckie A.J., Lindsay S.A., Jameson B., Hampson B., Holmes E.C., Beatty J.A. (2018). A Novel Hepadnavirus Identified in an Immunocompromised Domestic Cat in Australia. Viruses.

[19] Magnius L., Mason W.S., Taylor J., Kann M., Glebe D., Deny P., Sureau C., Norder H., Ictv Report C. (2020). ICTV Virus Taxonomy Profile: Hepadnaviridae. J. Gen. Virol..

[20] Lanave G., Capozza P., Diakoudi G., Catella C., Catucci L., Ghergo P., Stasi F., Barrs V., Beatty J., Decaro N. (2019). Identification of hepadnavirus in the sera of cats. Sci. Rep..

[21] Anpuanandam K., Selvarajah G.T., Choy M.M.K., Ng S.W., Kumar K., Ali R.M., Rajendran S.K., Ho K.L., Tan W.S. (2021). Molecular detection and characterisation of Domestic Cat Hepadnavirus (DCH) from blood and liver tissues of cats in Malaysia. BMC Vet. Res..

[22] Piewbang C., Wardhani S.W., Chaiyasak S., Yostawonkul J., Chai-In P., Boonrungsiman S., Kasantikul T., Techangamsuwan S. (2020). Insights into the genetic diversity, recombination, and systemic infections with evidence of intracellular maturation of hepadnavirus in cats. PLoS ONE.

[23] Pesavento P.A., Jackson K., Hampson T., Munday J.S., Barrs V.R., Beatty J.A. (2019). A Novel Hepadnavirus is Associated with Chronic Hepatitis and Hepatocellular Carcinoma in Cats. Viruses.

[24] Loomba R., Liang T.J. (2017). Hepatitis B Reactivation Associated With Immune Suppressive and Biological Modifier Therapies: Current Concepts, Management Strategies, and Future Directions. Gastroenterology.

[25] Lok A.S., McMahon B.J. (2009). Chronic hepatitis B: Update 2009. Hepatology.

[26] Capozza P., Lanave G., Diakoudi G., Stasi F., Ghergo P., Ricci D., Santo G., Arena G., Grillo I., Delle Donne E. (2021). A longitudinal observational study in two cats naturally-infected with hepadnavirus. Vet. Microbiol..

[27] Guidotti L.G., Isogawa M., Chisari F.V. (2015). Host-virus interactions in hepatitis B virus infection. Curr. Opin. Immunol..

[28] Bayton W.A., Westgarth C., Scase T., Price D.J., Bexfield N.H. (2018). Histopathological frequency of feline hepatobiliary disease in the UK. J. Small Anim. Pract..

[29] Desmet V.J., Gerber M., Hoofnagle J.H., Manns M., Scheuer P.J. (1994). Classification of chronic hepatitis: Diagnosis, grading and staging. Hepatology.

[30] Dandri M., Petersen J. (2016). Mechanism of Hepatitis B Virus Persistence in Hepatocytes and Its Carcinogenic Potential. Clin. Infect. Dis..

[31] Yang J.D., Kim W.R., Coelho R., Mettler T.A., Benson J.T., Sanderson S.O., Therneau T.M., Kim B., Roberts L.R. (2011). Cirrhosis is present in most patients with hepatitis B and hepatocellular carcinoma. Clin. Gastroenterol. Hepatol..

[32] Hammer A.S., Sikkema D.A. (1995). Hepatic neoplasia in the dog and cat. Vet. Clin. N. Am. Small Anim. Pract..

[33] Mason A., Wick M., White H., Perrillo R. (1993). Hepatitis B virus replication in diverse cell types during chronic hepatitis B virus infection. Hepatology.

[34] Shang Q., Zhang G., Xu C., Chen C.X., Yu J.G., Yan C.Y. (2001). Expression of hepatitis B virus antigen in brain tissue from liver cirrhosis patients with hepatitis B and its significance. Zhonghua Shi Yan He Lin Chuang Bing Du Xue Za Zhi.

[35] Pronier C., Guyader D., Jezequel C., Tattevin P., Thibault V. (2018). Contribution of quantitative viral markers to document hepatitis B virus compartmentalization in cerebrospinal fluid during hepatitis B with neuropathies. J. Neurovirol..

[36] Wang J., Huang H., Liu Y., Chen R., Yan Y., Shi S., Xi J., Zou J., Yu G., Feng X. (2020). HBV Genome and Life Cycle. Adv. Exp. Med. Biol..

[37] Li F., Wang Z., Hu F., Su L. (2020). Cell Culture Models and Animal Models for HBV Study. Adv. Exp. Med. Biol..

[38] Li H., Yan L., Shi Y., Lv D., Shang J., Bai L., Tang H. (2020). Hepatitis B Virus Infection: Overview. Adv. Exp. Med. Biol..

[39] Rein D.B., Stevens G.A., Theaker J., Wittenborn J.S., Wiersma S.T. (2012). The global burden of hepatitis E virus genotypes 1 and 2 in 2005. Hepatology.

[40] Ahmad I., Holla R.P., Jameel S. (2011). Molecular virology of hepatitis E virus. Virus Res..

[41] Purdy M.A., Harrison T.J., Jameel S., Meng X.J., Okamoto H., Van der Poel W.H.M., Smith D.B., Ictv Report C. (2017). ICTV Virus Taxonomy Profile: Hepeviridae. J. Gen. Virol..

[42] Panda S.K., Varma S.P. (2013). Hepatitis e: Molecular virology and pathogenesis. J. Clin. Exp. Hepatol..

[43] Panda S.K., Thakral D., Rehman S. (2007). Hepatitis E virus. Rev. Med. Virol..

[44] Sridhar S., Yip C.C.Y., Wu S., Cai J., Zhang A.J., Leung K.H., Chung T.W.H., Chan J.F.W., Chan W.M., Teng J.L.L. (2018). Rat Hepatitis E Virus as Cause of Persistent Hepatitis after Liver Transplant. Emerg. Infect. Dis..

[45] Andonov A., Robbins M., Borlang J., Cao J., Hatchette T., Stueck A., Deschambault Y., Murnaghan K., Varga J., Johnston L. (2019). Rat Hepatitis E Virus Linked to Severe Acute Hepatitis in an Immunocompetent Patient. J. Infect. Dis..

[46] Drexler J.F., Seelen A., Corman V.M., Fumie Tateno A., Cottontail V., Melim Zerbinati R., Gloza-Rausch F., Klose S.M., Adu-Sarkodie Y., Oppong S.K. (2012). Bats worldwide carry hepatitis E virus-related viruses that form a putative novel genus within the family Hepeviridae. J. Virol..

[47] Batts W., Yun S., Hedrick R., Winton J. (2011). A novel member of the family Hepeviridae from cutthroat trout (*Oncorhynchus clarkii*). Virus Res..

[48] Kumar Acharya S., Kumar Sharma P., Singh R., Kumar Mohanty S., Madan K., Kumar Jha J., Kumar Panda S. (2007). Hepatitis E virus (HEV) infection in patients with cirrhosis is associated with rapid decompensation and death. J. Hepatol..

[49] Kamar N., Bendall R., Legrand-Abravanel F., Xia N.S., Ijaz S., Izopet J., Dalton H.R. (2012). Hepatitis E. Lancet.

[50] Kamar N., Izopet J., Pavio N., Aggarwal R., Labrique A., Wedemeyer H., Dalton H.R. (2017). Hepatitis E virus infection. Nat. Rev. Dis. Primers.

[51] Kamar N., Izopet J., Dalton H.R. (2013). Chronic hepatitis e virus infection and treatment. J. Clin. Exp. Hepatol..

[52] Khuroo M.S., Khuroo M.S., Khuroo N.S. (2016). Hepatitis E: Discovery, global impact, control and cure. World J. Gastroenterol..

[53] Lole K., Bhukya P.L., Haldipur B., Liu D. (2019). Hepatitis E Virus. Handbook of Foodborne Diseases.

[54] Tei S., Kitajima N., Takahashi K., Mishiro S. (2003). Zoonotic transmission of hepatitis E virus from deer to human beings. Lancet.

[55] Smith D.B., Simmonds P., Jameel S., Emerson S.U., Harrison T.J., Meng X.J., Okamoto H., Van der Poel W.H.M., Purdy M.A., Members of the International Committee on the Taxonomy of Viruses Hepeviridae Study Group (2014). Consensus proposals for classification of the family Hepeviridae. J. Gen. Virol..

[56] Montagnaro S., De Martinis C., Sasso S., Ciarcia R., Damiano S., Auletta L., Iovane V., Zottola T., Pagnini U. (2015). Viral and Antibody Prevalence of Hepatitis E in European Wild Boars (*Sus scrofa*) and Hunters at Zoonotic Risk in the Latium Region. J. Comp. Pathol..

[57] Yugo D.M., Meng X.J. (2013). Hepatitis E virus: Foodborne, waterborne and zoonotic transmission. Int. J. Environ. Res. Public Health.

[58] Doceul V., Bagdassarian E., Demange A., Pavio N. (2016). Zoonotic Hepatitis E Virus: Classi fi cation, Animal Reservoirs and Transmission Routes. Viruses.

[59] Di Martino B., Di Profio F., Melegari I., Sarchese V., Robetto S., Marsilio F., Martella V. (2016). Detection of hepatitis E virus (HEV) in goats. Virus Res..

[60] Spahr C., Ryll R., Knauf-Witzens T., Vahlenkamp T.W., Ulrich R.G., Johne R. (2017). Serological evidence of hepatitis E virus infection in zoo animals and identification of a rodent-borne strain in a Syrian brown bear. Vet. Microbiol..

[61] Okamoto H., Takahashi M., Nishizawa T., Usui R., Kobayashi E. (2004). Presence of antibodies to hepatitis E virus in Japanese pet cats. Infection.

[62] Mochizuki M., Ouchi A., Kawakami K., Ishida T., Li T.C., Takeda N., Ikeda H., Tsunemitsu H. (2006). Epidemiological study of hepatitis E virus infection of dogs and cats in Japan. Vet. Rec..

[63] Liang H., Chen J., Xie J., Sun L., Ji F., He S., Zheng Y., Liang C., Zhang G., Su S. (2014). Hepatitis E virus serosurvey among pet dogs and cats in several developed cities in China. PLoS ONE.

[64] Dahnert L., Conraths F.J., Reimer N., Groschup M.H., Eiden M. (2018). Molecular and serological surveillance of Hepatitis E virus in wild and domestic carnivores in Brandenburg, Germany. Transbound. Emerg. Dis..

[65] Arankalle V.A., Joshi M.V., Kulkarni A.M., Gandhe S.S., Chobe L.P., Rautmare S.S., Mishra A.C., Padbidri V.S. (2001). Prevalence of anti-hepatitis E virus antibodies in different Indian animal species. J. Viral Hepat..

[66] Zhang W., Shen Q., Mou J., Gong G., Yang Z., Cui L., Zhu J., Ju G., Hua X. (2008). Hepatitis E virus infection among domestic animals in eastern China. Zoonoses Public Health.

[67] Liu J., Zhang W., Shen Q., Yang S., Huang F., Li P., Guo X., Yang Z., Cui L., Zhu J. (2009). Prevalence of antibody to hepatitis E virus among pet dogs in the Jiang-Zhe area of China. Scand J. Infect. Dis..

[68] Peralta B., Casas M., de Deus N., Martin M., Ortuno A., Perez-Martin E., Pina S., Mateu E. (2009). Anti-HEV antibodies in domestic animal species and rodents from Spain using a genotype 3-based ELISA. Vet. Microbiol..

[69] Lyoo K.S., Yang S.J., Na W., Song D. (2019). Detection of antibodies against hepatitis E virus in pet veterinarians and pet dogs in South Korea. Ir. Vet. J..

[70] Li Y., Qu C., Spee B., Zhang R., Penning L.C., de Man R.A., Peppelenbosch M.P., Fieten H., Pan Q. (2020). Hepatitis E virus seroprevalence in pets in the Netherlands and the permissiveness of canine liver cells to the infection. Ir. Vet. J..

[71] Kuno A., Ido K., Isoda N., Satoh Y., Ono K., Satoh S., Inamori H., Sugano K., Kanai N., Nishizawa T. (2003). Sporadic acute hepatitis E of a 47-year-old man whose pet cat was positive for antibody to hepatitis E virus. Hepatol. Res..

[72] Capozza P., Martella V., Lanave G., Beikpour F., Di Profio F., Palombieri A., Sarchese V., Marsilio F., La Rosa G., Suffredini E. (2021). A surveillance study of hepatitis E virus infection in household cats. Res. Vet. Sci..

[73] Khuroo M.S., Kamili S., Khuroo M.S. (2009). Clinical course and duration of viremia in vertically transmitted hepatitis E virus (HEV) infection in babies born to HEV-infected mothers. J. Viral Hepat..

[74] Meng X.J., Wiseman B., Elvinger F., Guenette D.K., Toth T.E., Engle R.E., Emerson S.U., Purcell R.H. (2002). Prevalence of antibodies to hepatitis E virus in veterinarians working with swine and in normal blood donors in the United States and other countries. J. Clin. Microbiol..

[75] Pavio N., Meng X.J., Renou C. (2010). Zoonotic hepatitis E: Animal reservoirs and emerging risks. Vet. Res..

[76] Kantala T., Kinnunen P.M., Oristo S., Jokelainen P., Vapalahti O., Maunula L. (2017). Hepatitis E Virus Antibodies in Finnish Veterinarians. Zoonoses Public Health.

[77] Mesquita J.R., Oliveira D., Rivadulla E., Abreu-Silva J., Varela M.F., Romalde J.L., Nascimento M.S. (2016). Hepatitis E virus genotype 3 in mussels (*Mytilus galloprovinciallis*), Spain. Food Microbiol..

